# Supplementary material for: Follow-Up of Men Who Have Undergone Focal Therapy for Prostate Cancer with HIFU—A Real-World Experience
Source: J Clin Med. 2023 Nov 14;12(22):7089. doi: 10.3390/jcm12227089 (PMC10672492; doi:10.3390/jcm12227089)
Supplement: Supplementary file 1 [file jcm-12-07089-s001.zip › jcm-2655824-supplementary.pdf]

Table S1. Pre and post HIFU biopsy results in recurrence patients.

pre HIFU		post HIFU			
lesion_1	lesion_2	lesion_1	lesion_2	outfield_1	outfield_2
3+3	3+3	3+3	3+4	3+3	
3+4		3+4		4+4	3+4
3+4				3+4	4+4
3+3				3+4	3+3
3+3				3+3	
3+3	3+3	3+3	3+3		
3+4		3+3			
3+3	3+3	3+4	3+3	3+3	
3+3	3+3	3+3	3+4	3+3	
3+3		3+4		3+3	4+5
3+4				4+4	3+4
3+4		3+3		3+3	3+3
3+3		3+3			
3+3		3+3			
3+3		3+3			
3+4		3+3			
4+4		3+3			
3+3	3+3		3+3		
